# Isolation and identification of the *cis*-abienol degrading strain *Klebsiella oxytoca* T2L and its aroma products

**DOI:** 10.1038/s41598-025-07179-z

**Published:** 2025-07-01

**Authors:** Li Han, Xinlong Zhang, Hang Xu, Gaolei Xi, Ke Wang, Haoyang Chen, Wenyuan Qi, Liuke Zhang, Shen Huang

**Affiliations:** 1 Zhengzhou University of Light Industry, 136 Science Avenue, Zhengzhou High tech Zone, Zhengzhou, 450002 China; 2 Technology Center for China Tobacco Henan Industrial Limited Company, Zhengzhou, 450000 Henan People’s Republic of China

**Keywords:** *Klebsiella oxytoca*, Biodegradation, *cis*-abienol, Aroma compounds, Biotechnology, Microbiology

## Abstract

*Cis*-abienol, a naturally occurring labdanum diterpenoid, is believed to be an essential precursor of ambergris substances and is found on the surface of many plants. In this study, the strain *Klebsiella oxytoca* T2L was isolated from the soil of oriental tobacco and demonstrated effective degradation of *cis*-abienol. The strain was identified through morphological characteristics and 16 S rDNA sequence analysis. Under culture conditions of 30 °C, pH 7.0, 0.5 mg/mL ammonium sulfate, and 1 mg/mL *cis*-abienol—the degradation rate of *cis*-abienol by strain T2L reached 68.2% after 96 h. The influence of various carbon and nitrogen sources on the degradation effect of *cis*-abienol by T2L was examined. Additionally, the impact of pH and temperature on cell growth and *cis*-abienol degradation was also analyzed. GC-MS analysis indicated that the degradation of *cis*-abienol by strain T2L generated several potential aroma chemicals, including (+/-)-ambreinolide, sclareol, an analog of amberonne (isomer 3), and an analog of sclaral (sclareolide lactol), which resembled the composition of natural ambergris. This study provides a theoretical foundation for further research on the biodegradation of *cis*-abienol to high-value aroma compounds.

**Supplementary Information:**

The online version contains supplementary material available at 10.1038/s41598-025-07179-z.

## Introduction

*Cis*-abienol, a naturally occurring labdanum diterpenoid^1^, has a molecular weight of 290.48. It is one of the exudates found on the surface of plants^2^; however, its contents are scarce and limited to a few plants. Initially discovered in the oil resin of Canadian fir, *cis*-abienol has been subsequently found in the essential oils of coniferous plants^3,4^, in the tuber of Compositaceae snow lily, and in the glandular hairs of tobacco^5–7^. Studies have demonstrated that *cis*-abienol possesses various biological functions, including insect resistance and disease resistance^8–10^, and it helps prevent other microbial infections^11–13^. For instance, *cis*-abienol has been used as an inducer to effectively control bacterial wilt in tomatoes and other plants^14^.

Despite its limited presence in plants, *cis*-abienol plays a crucial role in aroma properties^15–17^. It has been identified as an essential precursor of ambergris substances^18–20^, which are among the most precious spices in the world today. Ambergris is known for its lasting aroma, excellent harmonization, and strong fixative properties^21,22^. Currently, ambergris is primarily used as a tincture in the formulation of high-end and expensive perfumes, holding an indispensable position in fragrance creation. Among ambergris substitutes, ambrox^23^ and norambreinolide^24^ are prominent examples.

Although *cis*-abienol has been identified as a precursor for the chemical synthesis of ambergris-like substances, research on the degradation or transformation of *cis*-abienol into such products remains limited. Huang Tingting et al.^25^ summarized the metabolic regulation mechanism of labdanum diterpenoids and illustrated that *cis*-abienol could produce compounds such as sclareol, ambrox, and norambreinolide through redox reactions. Tadaharu Hieda et al.^26^ isolated a strain, JTS-131, capable of catalyzing *cis*-abienol degradation, yielding products such as (12Z)-labda-12,14-dien-18-01 and 12(Z)-labda-12,14-dien-18-oic acid and its methyl ester. Mohamad I. Farbood^27^ used two bacterial strains, *Cryptococcus albidus* ATCC20918 and *Hyphozyma roseonigra* ATCC20624, to transform sclareol into sclareolide and sclareol glycol. Similarly, S. A. Kouzi et al.^28^ investigated the microbial metabolism of sclareol using *Bacillus cereus* UI-1477, identifying seven metabolites.

In this study, a novel microbial strain capable of degrading *cis*-abienol as the sole carbon source to generate ambergris-like substances has been isolated. The degraded products were identified through GC-MS, and various growth parameters affecting the degradation rate of *cis*-abienol were optimized. This research provides a theoretical basis for the biodegradation of *cis*-abienol into valuable ambergris-like products.

## Results

### Isolation and identification of *cis*-abienol degrading bacteria

**Fig. 1:**
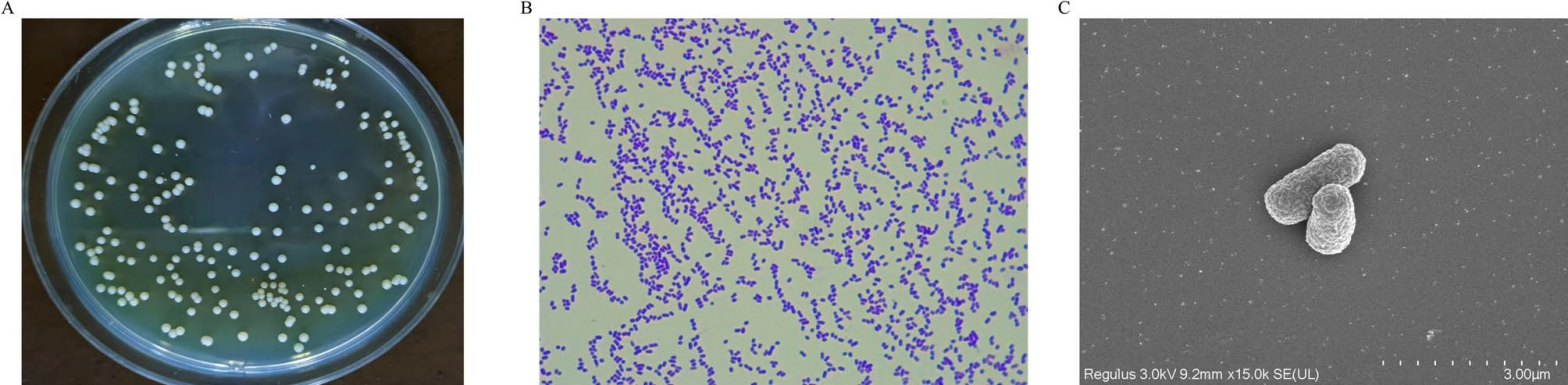
Morphological characteristics of the screened strain T2L. (A) Colony morphology. (B) Gram staining results. (C) Scanning electron microscopy results.

A strain capable of degrading *cis*-abienol was isolated using *cis*-abienol as the sole carbon source from the soil of aromatic tobacco. The strain was designated as T2L. Colonies of T2L were observed to form white, round spots with distinct transparent edges and a solid center. These colonies were opaque, lacked mycelia, and had a smooth appearance (Fig. 1A). Gram staining revealed that T2L was gram-positive (Fig. 1B). Scanning electron microscopy (15,000× magnification) showed that the strain consisted of short, rod-like cells measuring 7–9 μm in length and 18–20 μm in width. The cells lacked flagella and exhibited wrinkled surfaces (Fig. 1C).

**Fig. 2:**
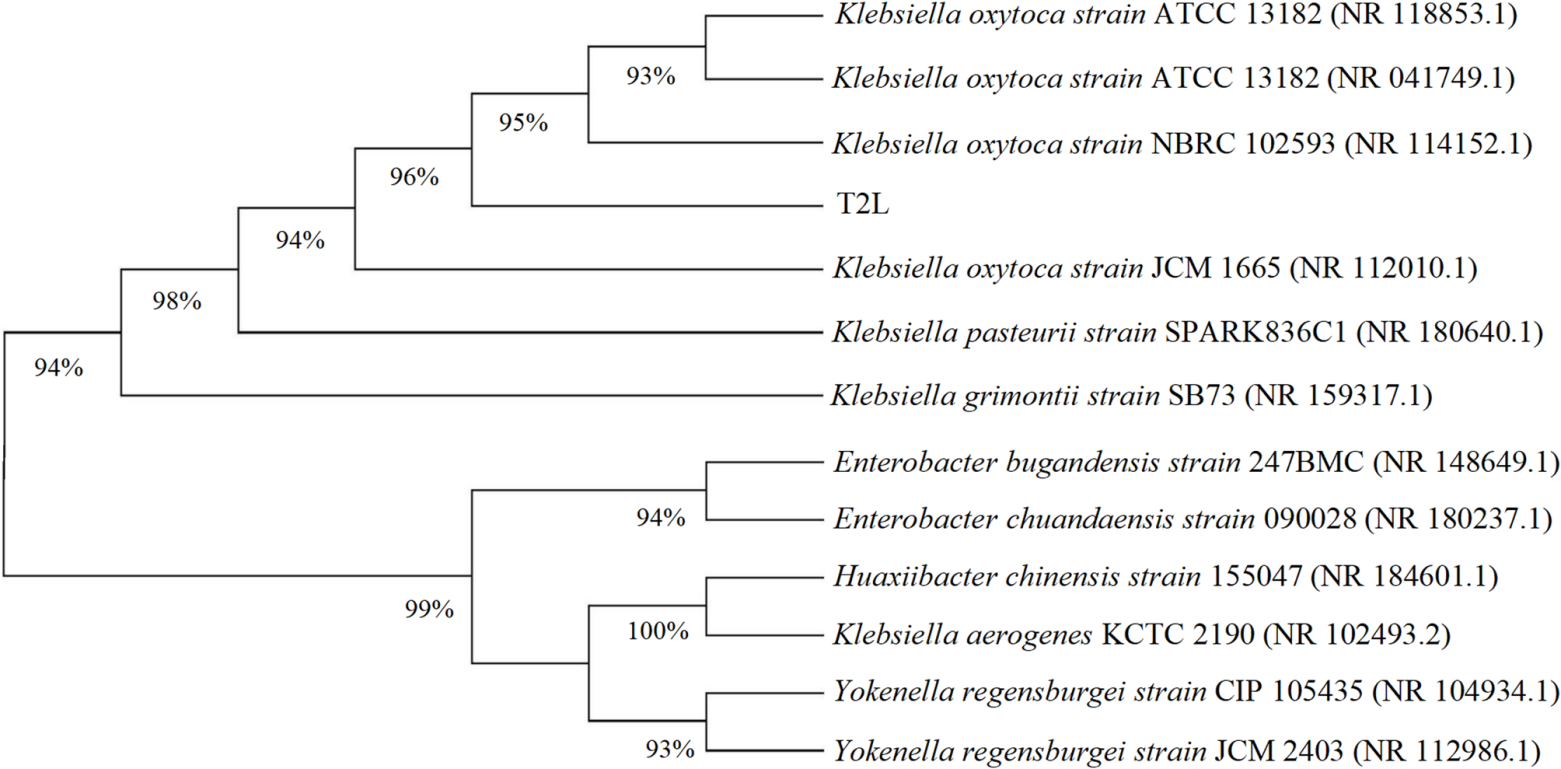
Phylogenetic tree construction using MEGA software based on the 16 S rDNA sequence.

Following purification, the strain was identified by 16 S rDNA technique, revealing a sequence length of 1,404 bp. BLAST homology analysis showed that the 16 S rDNA sequence of T2L shared 99.93% similarity with *Klebsiella oxytoca* M0572 and *Klebsiella sp.* HNDS8, and 99.79% similarity with *Klebsiella pasteurii* SPARK836C1. Phylogenetic analysis indicated that T2L formed a distinct species lineage within the *K. oxytoca* cluster. Although closely related, T2L occupied a separate branch in the phylogenetic tree, leading to its identification as *K. oxytoca* (Fig. 2).

### The growth and degradation conditions of T2L in a medium containing *cis*-abienol

**Fig. 3:**
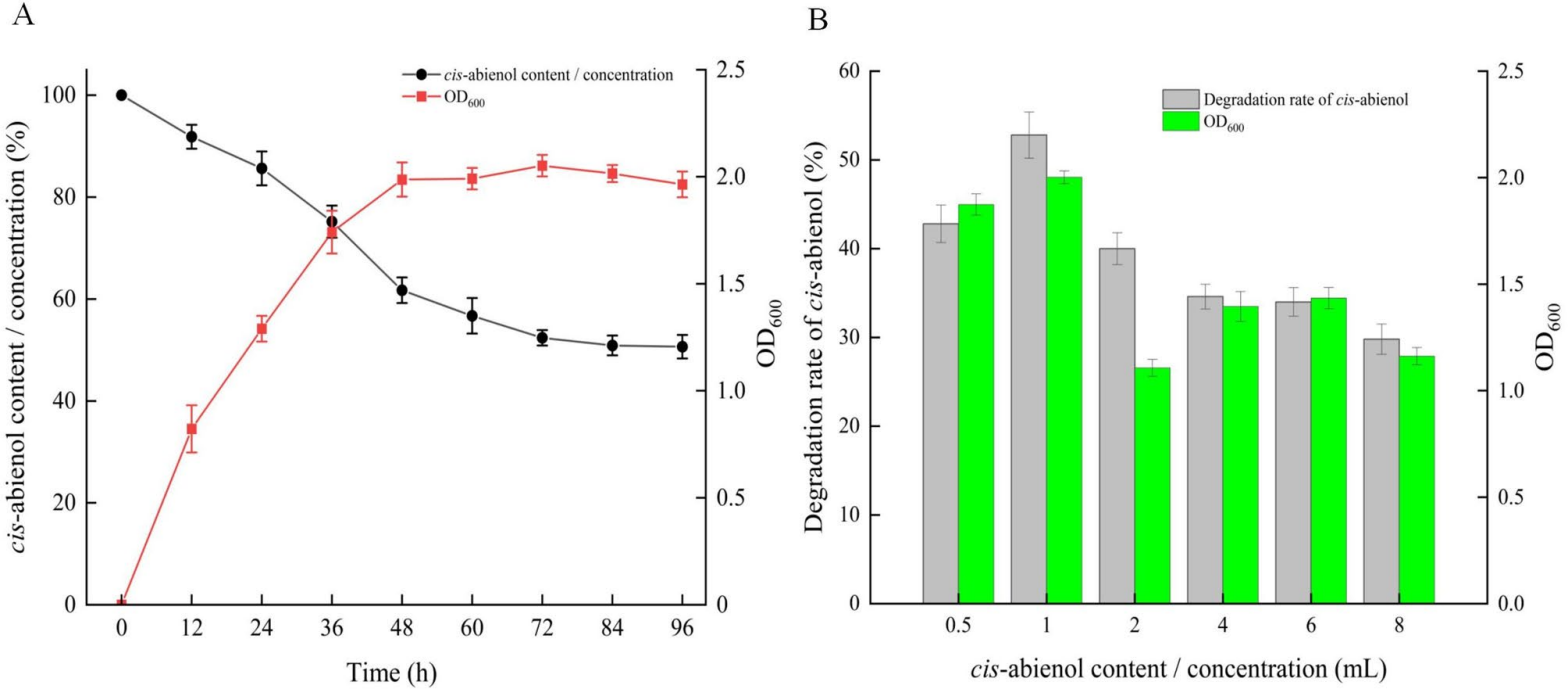
Growth conditions and degradation rate of *cis*-abienol by T2L. (A) Cell growth of T2L in medium containing 1 mg/mL cis-abienol. (B) Degradation rates of *cis*-abienol at different initial concentrations.

After screening for the *cis*-abienol-degrading strain, T2L was inoculated into a medium containing *cis*-abienol at 1 mg/mL as the sole carbon source. Strain T2L exhibited rapid growth during the first 48 h, entering the logarithmic phase with an OD_600_ of 1.9. After 48 h, the strain reached the stationary phase, with bacterial content remaining stable. Concurrently, the *cis*-abienol content in the medium decreased, dropping sharply between 24 and 48 h. By 72 h, the relative content of *cis*-abienol had stabilized at 49.4%. After 96 h, the degradation rate slowed further, resulting in a relative *cis*-abienol content of 48.6% (Fig. 3A). The negative control used was the same medium containing *cis*-abienol at 1 mg/mL concentrations without T2L. The results indicated that cis-abienol remained stable throughout the entire fermentation process (Fig. S1).

To assess the effect of *cis*-abienol concentration on its degradation process, experiments were performed with various initial concentrations of *cis*-abienol. Specifically, at an initial concentration of 1 mg/mL, the degradation rate was found to be the highest, achieving 54.6%. At an initial concentration of 8 mg/mL, the degradation rate decreased significantly to 29.7% (Fig. 3B). Interestingly, at 0.5 mg/mL, the degradation rate was slightly lower than at 1 mg/mL, reaching 42.3%. Multiple repeats confirmed these results, indicating a consistent trend.

### Analysis of possible metabolites formed from *cis*-abienol during fermentation with the strain T2L

**Fig. 4:**
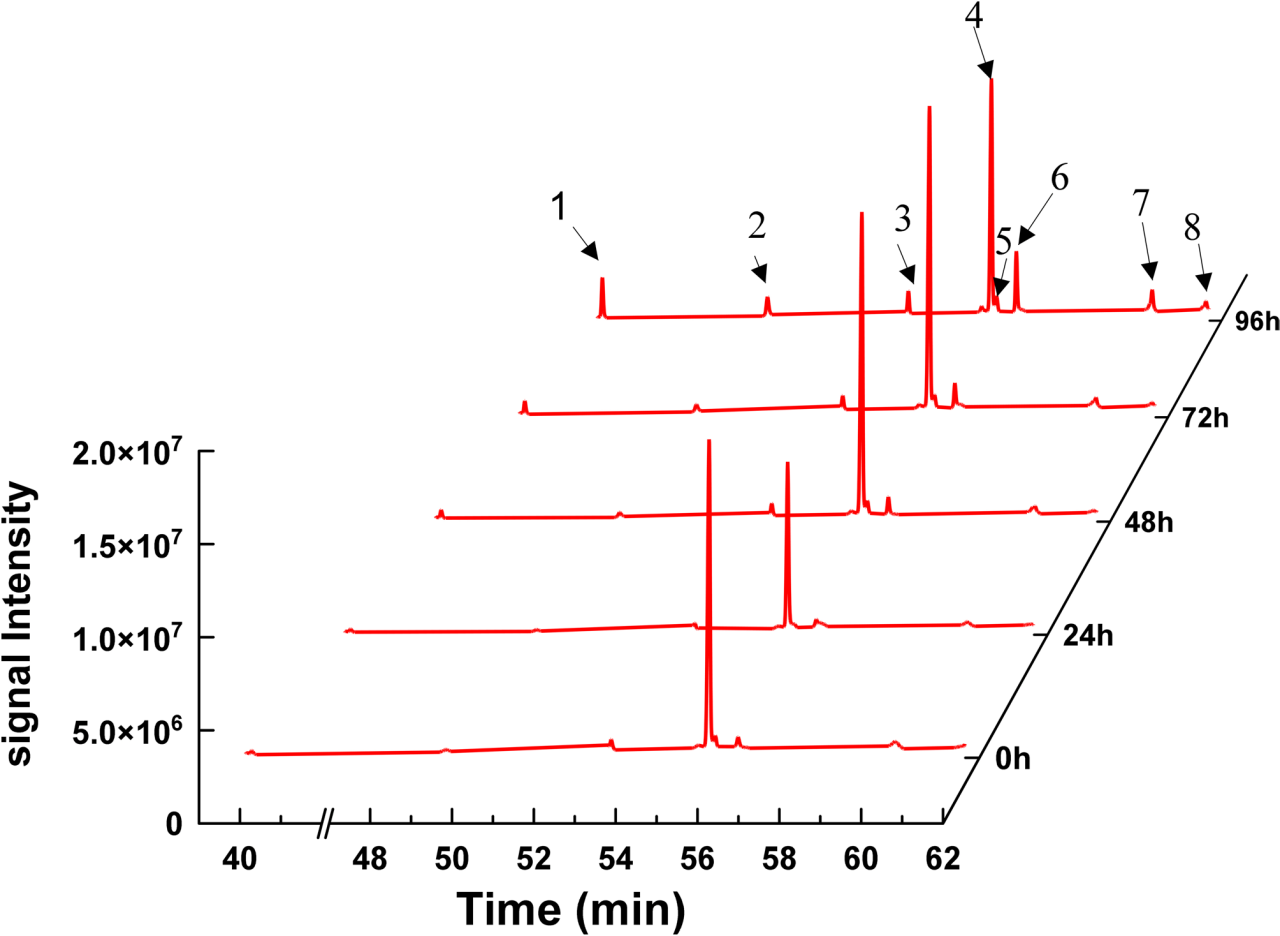
GC-MS analysis of fermentation products of T2L using *cis*-abienol as the sole carbon source. 1: *n*-Nonenylsuccinic anhydride; 2: Sclaral (sclareolide lactol); 3: Amberonne (isomer 3) analog; 4: Sclareol analog; 5: 15-Isobutyl-(13.alpha.H)-isocopalane; 6: (3R)-3,7-Dihydroxy-9,11-eremophiladien-8-one, 3-acetate; 7: 2,5,5,8a-Tetramethyl-4-methylene-4a,5,6,7,8,8a-hexahydro-4 H-chromene; 8: (+/-)-Ambreinolide. The peak ion profile of the compounds on MS was supplemented in Fig. S4.

GC-MS analysis was performed to identify the degradation products of *cis*-abienol by strain T2L during fermentation. Several chemicals were detected as possible degradation products when T2L utilized *cis*-abienol as the sole carbon source (Fig. 4). After 24 h, a new compound 6, potentially identified as (3R)-3,7-dihydroxy-9,11-eremophiladien-8-one 3-acetate, was detected and its concentration increased to 66.6 µg/mL by 96 h. At 48 h, a compound 7, potentially identified as 2,5,5,8a-tetramethyl-4-methylene-4a,5,6,7,8,8a-hexahydro-4 H-chromene was detected, reaching 30 µg/mL by 96 h. Another two possible compounds 8 and 5, (+/-)-ambreinolide and 15-isobutyl-(13.alpha.H)-isocopalane, were detected after 72 h, with concentrations increasing to 10.9 µg/mL and 23.1 µg/mL, respectively. Minor amounts of manoyl oxide, manool, and ambrial were also detected after 96 h according to the GC-MS analysis (Fig. 4; Table 1).

The *cis*-abienol utilized in the experiments was obtained commercially and contained various impurities as determined by GC-MS analysis, including amberonne (an isomer of cis-abienol), sclareol analog, sclaral, and n-nonenylsuccinic anhydride. During fermentation, the concentrations of amberonne analog (compound 3), sclaral (compound 2), and n-nonenylsuccinic anhydride (compound 1) increased to 40.7, 28.7, and 42.4 µg/mL, representing 10.3-, 14.35-, and 10.6-fold increases, respectively, compared to the initial levels. In contrast, the concentration of sclareol analog (compound 4) remained relatively unchanged.

**Table 1:** GC-MS analysis of potential chemicals related to *cis*-abienol degradation by T2L.

Possible product	Content (ug/mL)				
	0 h	24 h	48 h	72 h	96 h
(+/-)-Ambreinolide	-	-	-	1.5	10.9
Manoyl oxide	-	-	-	-	1.8
Manool	-	-	-	-	0.5
Ambrial	-	-	-	-	1.1
Amberonne (isomer 3) analog	3.6	1.8	8.0	6.2	40.7
Sclareol analog	254.5	141.2	294.7	300.4	262.6
Sclaral (sclareolide lactol)	2.0	2.0	9.1	13.0	28.7
n-Nonenylsuccinic anhydride	4.0	3.1	9.1	12.4	42.4
(3R)-3,7-Dihydroxy-9,11- eremophiladien-8-one, 3-acetate	-	8.7	15.7	28.2	66.6
15-Isobutyl- (13.alpha.H)-isocopalane	-	-	-	3.3	23.1
2,5,5,8a-Tetramethyl-4-methylene- 4a,5,6,7,8,8a-hexahydro-4 H-chromene	-	-	8.6	19.4	30.0

Note: “-” indicates no detection.

### The influence of different sources of carbon and nitrogen on *cis*-abienol degradation

**Fig. 5:**
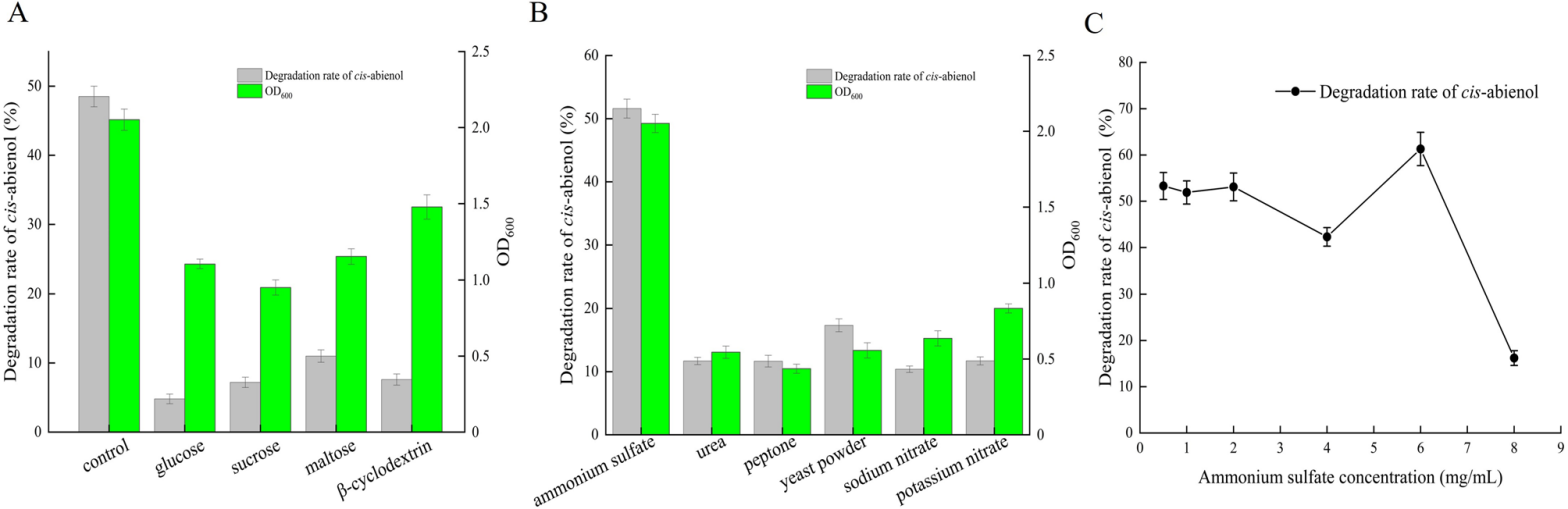
The effect of carbon and nitrogen sources on the degradation rate of *cis*-abienol by T2L. (A) Effect of different carbon sources. (B) Effect of different nitrogen sources. (C) Effect of ammonium sulfate concentration.

To determine the effects of various carbon sources on the degradation of *cis*-abienol by strain T2L, several carbon sources at a concentration of 1 mg/mL were added to the medium separately alongside *cis*-abienol. The results indicated that the addition of other carbon sources was detrimental to *cis*-abienol degradation. Glucose, sucrose, and maltose significantly inhibited the growth of T2L, with OD_600_ values decreasing by 46.34%, 53.65%, and 43.81%, respectively. Similarly, β-cyclodextrin reduced cell growth in the inorganic medium by 27.97%. Moreover, the degradation rate of *cis*-abienol was also negatively affected by the addition of these carbon sources. The degradation rates decreased significantly by 90.1% with glucose, 85.15% with sucrose, 77.3% with maltose, and 84.32% with β-cyclodextrin (Fig. 5A). These findings suggest that the addition of alternative carbon sources is not beneficial for either cell growth or *cis*-abienol degradation by T2L in an inorganic medium.

Following the analysis of carbon sources, the effects of different nitrogen sources on *cis*-abienol degradation were also examined. The inorganic medium initially contained 0.5 mg/mL of ammonium sulfate as the nitrogen source. To evaluate the influence of other nitrogen sources, 0.5 mg/mL of urea, peptone, yeast powder, sodium nitrate, or potassium nitrate was used to replace ammonium sulfate. The results showed that all these nitrogen sources significantly affected both cell growth and *cis*-abienol degradation. The addition of urea, peptone, yeast powder, and sodium nitrate caused reductions in cell growth and *cis*-abienol degradation of more than 50%. Potassium nitrate also had a significant inhibitory effect, reducing cell growth and degradation by 59.32% and 77.32%, respectively (Fig. 5B). Among all tested nitrogen sources, ammonium sulfate yielded the best results for cell growth and *cis*-abienol degradation.

Since ammonium sulfate was the most effective nitrogen source for T2L in degrading cis-abienol, the optimal concentration of ammonium sulfate for *cis*-abienol degradation was investigated. Ammonium sulfate concentrations ranging from 0.5 to 8 mg/mL were tested. The peak degradation rate of *cis*-abienol was achieved at 61.3% when the concentration of ammonium sulfate was 6 mg/mL. However, at a concentration of 8 mg/mL, the degradation rate sharply decreased to 16.2% (Fig. 5C). The optimal concentration of ammonium sulfate was therefore determined to be 6 mg/mL.

### The influence of pH and temperature on cell growth of T2L and *cis*-abienol degradation

**Fig. 6:**
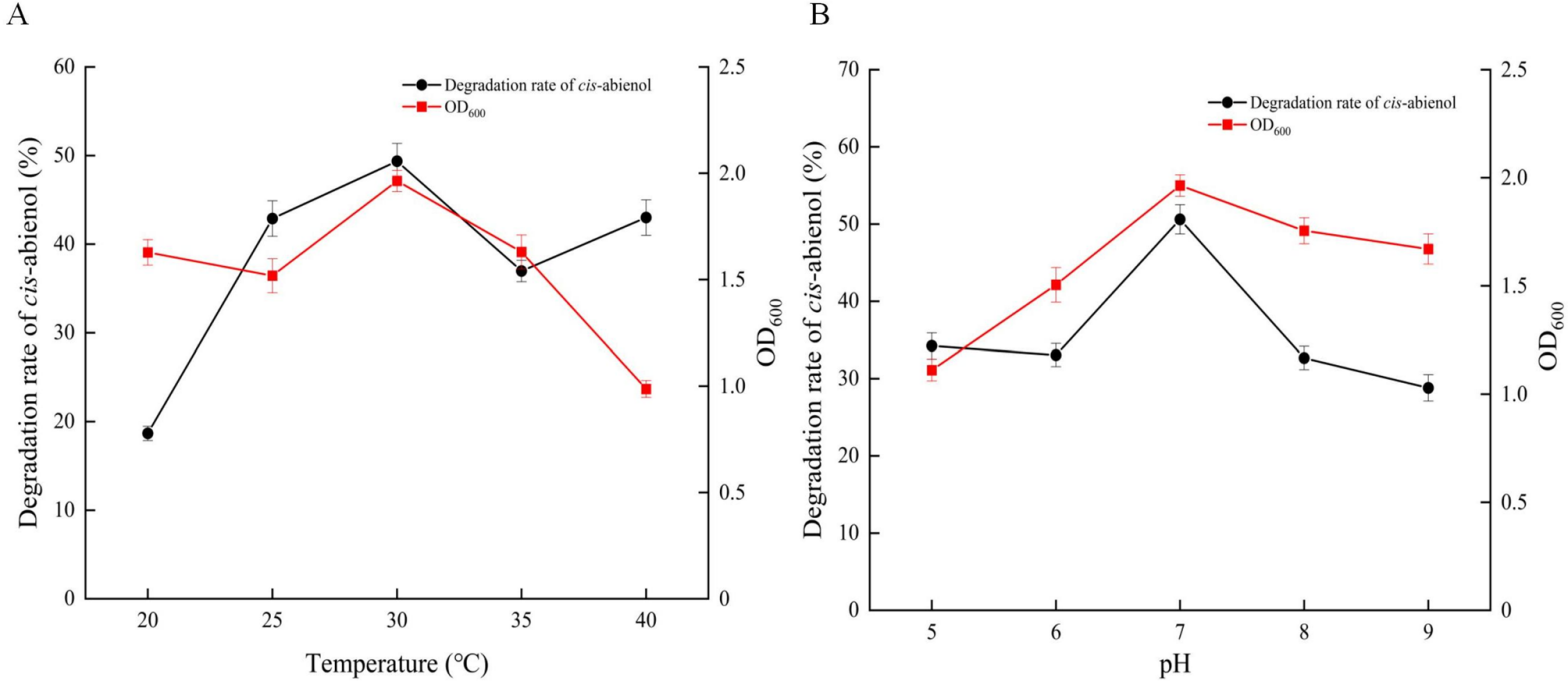
The effect of different temperatures and pH on the degradation of *cis*-abienol by strain T2L. (A) Effect of temperature. (B) Effect of pH.

Further experiments were performed to evaluate the impact of different temperatures and pH levels on the growth of strain T2L and its capacity to degrade cis-abienol. The T2L strain was cultured in a medium containing 1 mg/mL of *cis*-abienol at temperatures of 20 °C, 25 °C, 30 °C, 35 °C and 40 °C, all with a constant rotation speed of 150 rpm. As shown in Fig. 6A, the highest cell OD_600_ value of approximately 2.0 was achieved at 30 °C, corresponding to the highest degradation rate of *cis*-abienol at 51.4%. Both cell growth and *cis*-abienol degradation declined as temperatures increased beyond 30 °C. These results identify 30 °C as the optimal temperature for T2L to degrade *cis*-abienol in the inorganic medium.

The impact of pH levels ranging from 5 to 9 on the growth and degradation rate of *cis*-abienol by strain T2L was also investigated. Initially, the stability of *cis*-abienol was examined in a medium with varying pH levels as a negative control. The findings revealed that *cis*-abienol is stable at pH 5 and pH 9 (Fig. S2). Subsequently, the pH value of the fermentation medium was tested, which contained strain T2L, *cis*-abienol, glucose as the carbon source, and different nitrogen sources like ammonium sulfate or peptone. It was observed that the pH value remained consistent throughout the entire experiment (Fig. S3). The results showed that the optimal pH for *cis*-abienol degradation was 7.0, achieving a degradation rate of approximately 52.9%. Furthermore, the optimal pH for cell growth was also found to be 7.0. Deviations from this pH value, either below or above 7.0, negatively affected both cell growth and the degradation rate of *cis*-abienol. Interestingly, at a pH of 9.0, the degradation rate of *cis*-abienol reached 28.82%, although it remained lower compared to the results at pH 7.0 (Fig. 6B) .

## Discussion

In this study, the *cis*-abienol-degrading strain T2L was successfully isolated and identified using cell morphology, cell size analysis, and 16 S rDNA sequencing. BLAST homology analysis through the NCBI GenBank database confirmed that strain T2L is a member of *K. oxytoca*. Previous studies have shown that species within this genus can degrade nitrobenzene and 4-chloronitrobenzene and improve the salt-alkali tolerance of corn seedlings, highlighting their potential applications in agriculture and environmental management^29,30^. In the present study, *K. oxytoca* T2L degraded 68.2% of *cis*-abienol (1 mg/mL) within 96 h, producing several substances, including labdanum and ambergris-like compounds, thereby confirming its potential as a *cis*-abienol-degrading strain.

The results displayed that ammonia sulfate was much better than other nitrogen sources, including organic nitrogen and certain inorganic nitrogens. It has been reported that certain nitrogen in ammonium salts can be directly absorbed and utilized by bacterium, while nitrogen in nitrate or organic nitrogen needs to be converted to ammonia prior to absorption^31^. Additionally, we speculate that sulfate might be a beneficial substance for some enzymes in strain T2L.

**Fig. 7:**
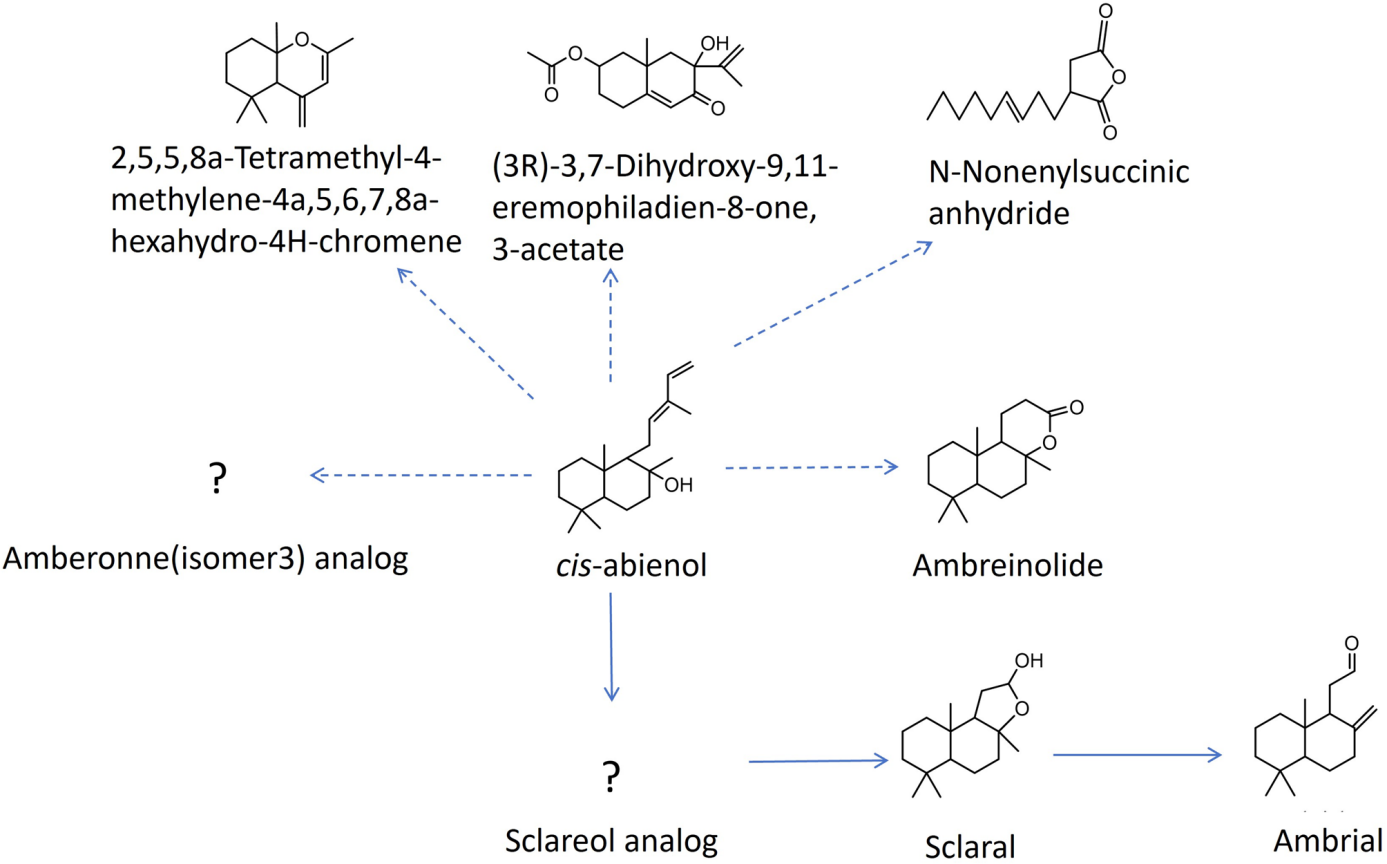
Prediction of the chemicals involved in the *cis*-abienol degradation process using strain T2L.

It has been reported by Tingting Huang et al.^25^ that *cis*-abienol can be transformed into sclareol. In our experiment, however, a potential sclareol analog was detected using GC-MS in comparison to the standard sclareol. The concentration of this analog initially decreased and then increased after 72 h. This could be attributed to the similarity between *cis*-abienol and the sclareol analog. The detected chemical sclareol analog is present in commercial *cis*-abienol. We speculate that the sclareol analog was initially consumed by the cell and subsequently increased, potentially due to the biodegradation of *cis*-abienol by strain K. oxytoca T2L. Furthermore, after 96 h, possible chemicals such as (+/-)-ambreinolide, sclaral (sclareolide lactol) and amberonne (isomer 3) analog were detected. Studies suggest that *cis*-abienol^32^ and sclareol^33^ can be transformed into nor-ambreinolide^34^, with sclareol being the more probable precursor^35^. Combining the chemical reactions from *cis-abienol* to ambergris-like chemicals with published articles, it is speculated that *K. oxytoca* T2L transforms *cis*-abienol into a sclareol analog, which is then converted into (+/-)-ambreinolide, sclaral, and ambrial. For the amberonne (isomer 3) analog, no relevant studies are available and its specific degradation pathway remains unclear. Other major products such as (3R)-3,7-dihydroxy-9,11-eremophiladien-8-one,3-acetate and n-nonenylsuccinic anhydride are presumed to be metabolic byproducts generated during the utilization of *cis*-abienol by the strain. The predicted degradation pathway of *cis*-abienol by strain T2L is summarized in Fig. 7.

Strain T2L effectively degrades *cis*-abienol, producing several ambergris-like products, thus providing a valuable microbial resource for studying ambergris synthesis and *cis*-abienol degradation. Products such as (+/-)-ambreinolide, ambrial, and amberonne (isomer 3) share similarities with natural ambergris and exhibit an amber-like aroma, making them key intermediates in the synthesis of ambrox^36–38^. Sclaral (sclareolide lactol), presumed to be an oxidation product of sclareol, is not currently available in domestic or international markets.

## Conclusion

*K. oxytoca* T2L was identified as a strain capable of effectively degrading *cis*-abienol. The strain degraded 68.2% of 1 mg/mL *cis*-abienol within 96 h. Optimal growth and degradation conditions for *K. oxytoca* T2L were determined to be 30 °C, pH 7, 6 mg/mL ammonium sulfate, and 1 mg/mL *cis*-abienol. Gas chromatography-mass spectrometry (GC-MS) analysis of the degradation products revealed the formation of several aroma compounds, including (3R)-3,7-dihydroxy-9,11-eremophiladien-8-one,3-acetate, n-nonenylsuccinic anhydride, amberonne (isomer 3), (+/-)-ambreinolide, ambrial, sclaral (sclareolide lactol), and sclareol analog. This research provides a theoretical basis for the biodegradation of *cis*-abienol into valuable aroma compounds.

## Materials and methods

### Isolation of *cis*-abienol-degrading bacteria and the culture medium

Soil samples were collected from oriental tobacco fields in Zhengzhou, Henan Province, China, and transported to the laboratory for bacterial isolation. A 1 g soil sample was added to 100 mL sterile water in a conical flask under aseptic conditions. The mixture was soaked and shaken for approximately 1 h. From this suspension, 1% of the bacterial source was enriched in Luria–Bertani (LB) medium at 150 rpm/min and 30 °C for 6 h. Once the medium became slightly turbid, indicating enrichment, 1% of the enriched culture (diluted from 10⁻¹ to 10⁻⁶) was spread onto selective plates containing *cis*-abienol as the sole carbon source^39^.

The composition of the selective inorganic medium was as follows: K_2_HPO_4_ (1 g/L), MgSO_4_·7 H₂O (0.5 g/L), FeSO_4_·7 H₂O (0.005 g/L), NaCl (0.5 g/L), KH₂PO_4_ (0.65 g/L), MnSO_4_ (0.001 g/L), (NH_4_)_2_SO_4_ (0.5 g/L), CaCl_2_·2 H₂O (0.1 g/L), and Na_2_MoO_4_·2H_2_O (0.005 g/L). To screen for *cis*-abienol-degrading strains, 1 mg/mL *cis*-abienol was added to the medium. Plates were incubated at 30 °C for 1–2 days. Colonies appearing on the plates were isolated and cultured in an LB medium. Isolated strains were preserved in 60% (w/v) glycerol at − 80 °C. The fermentation medium used to evaluate *cis*-abienol degradation consisted of a selective inorganic medium with variable carbon or nitrogen sources, depending on experimental requirements.

### Molecular identification of T2L strain by 16 S rDNA and morphology observation by scanning electron microscopy

Colony morphology and cell shape were examined using high-resolution cold-field emission scanning electron microscopy (Hitachi, Regulus 8100). Genomic DNA was extracted using a bacterial genomic DNA rapid extraction kit (TIANamp Bacteria DNA Kit, TIANGEN BIOTECH, Beijing, China). The 16S rDNA was amplified using the primer pairs 5’-AGAGTTTGATCCTGGCTCAG-3’ and 5’-GGTTACCTTGTTACGACTT-3’, synthesized by Personalbio Technology (Shanghai) Co., Ltd. The PCR reaction yielded a 1,424 bp fragment, which was purified and sequenced by Personalbio Technology (Shanghai) Co., Ltd.

Phylogenetic analysis was conducted using MEGA 5.1 software, with a phylogenetic tree constructed based on the 16 S rDNA sequence. The neighbor-joining method was used, with 1,000 bootstrap replicates.

### Quantification method for *cis*-abienol

Quantification of *cis*-abienol was performed using standard *cis*-abienol solutions with varying concentrations analyzed by ultra-performance liquid chromatography (UPLC). The UPLC conditions were as follows^40–42^: the chromatographic column was a WATERS XBridge C18 Column (4.6 × 250 mm, 5 μm). The mobile phase consisted of methanol (90%), water (8%), and acetic acid (2%). The mobile phase was filtered through a 0.22 μm organic microporous filter membrane, and bubbles were removed by ultrasonic agitation. The column temperature was maintained at 30 °C, and detection was performed at a wavelength of 237 nm using a diode-array detector. The mobile phase flow rate was set at 1 mL/min, and the injection volume was 5 µL.

### GC-MS analysis of biodegradation products of *cis*-abienol

To analyze the biodegradation products of *cis*-abienol, 15 mL of fermentation solution was combined with an equal volume of analytical-grade dichloromethane. The mixture was subjected to vortex extraction at 2,500 rpm for 10 min and then centrifuged at 10,000 rpm for 10 min. The lower organic phase was collected, and the extraction steps were repeated three times. The combined organic phases were dried over anhydrous sodium sulfate and left to stand overnight. Subsequently, 100 µL of 6-dichlorotoluene internal standard solution was added, and the solution was concentrated to 1 mL at atmospheric pressure. The final solution was filtered through a 0.22 μm organic filter membrane and transferred to a chromatographic vial for analysis^43^.

*GC conditions:*

The GC column used was HP-5MS 5% PhenylMethylSilox (30 m × 0.25 mm × 0.25 μm). The sample inlet temperature was set at 280 °C. High-purity helium served as the carrier gas at a flow rate of 1 mL/min. The temperature program was as follows: Initial temperature of 50 °C, maintained for 3 min. Increase to 130 °C at 6 °C/min, held for 3 min. Further increase to 180 °C at 4 °C/min, held for 2 min. Final increase to 280 °C at 3 °C/min, maintained for 5 min. The flow split ratio was set at 5:1, and the injection volume was 1 µL. The GC-MS analysis was performed using full-spectrum scanning.

MS analysis conditions: Transfer line temperature: 280 °C. Ion source temperature: 230 °C. Quadrupole electrode temperature: 150 °C. Ionization mode: 70 eV electron impact. Reaction lag time: 7 min. Mass-to-charge ratio (m/z) scan range: 50 to 700.

### Measurement of cell growth of T2L strain in medium with *cis*-abienol as the sole carbon source

A single colony was picked from the LB medium plate and inoculated into 3 mL of LB liquid medium. The culture was incubated overnight at 150 rpm and 30 °C. The seed culture was then transferred into 20 mL of fermentation medium with an initial OD_600_ of 0.1. The fermentation was carried out at 150 rpm and 30 °C. Bacterial growth (OD_600_) was measured using a UV-Visible Spectrophotometer (UV-1500, Shanghai Macylab Instrument Co., Ltd).

To evaluate the effect of pH on bacterial growth and *cis*-abienol degradation, the pH of the fermentation medium was adjusted to 5.0, 6.0, 7.0, 8.0, or 9.0 using 0.1 M HCl or 0.1 M NaOH solutions.

## Electronic supplementary material

Below is the link to the electronic supplementary material.

Supplementary Material 1

## Data Availability

All sequencing reads obtained in this study have been deposited in the GenBank database (www.ncbi.nlm.nih.gov/genbank/) under accession numbers PV023313. The datas generated or analyzed during the current study are included in this article. The datasets generated or analyzed during the current study are available from the corresponding author on reasonable request.
