# Supplementary material for: Isolation and identification of the cis-abienol degrading strain Klebsiella oxytoca T2L and its aroma products
Source: Sci Rep. 2025 Jul 1;15:21824. doi: 10.1038/s41598-025-07179-z (PMC12219735; doi:10.1038/s41598-025-07179-z)
Supplement: Supplementary file 1 — Supplementary Material 1 [file 41598_2025_7179_MOESM1_ESM.docx]

**Isolation and Identification of the *Cis*-Abienol-Degrading *Klebsiella oxytoca* T2L Strain and its Aroma Products**

1. Stability of *cis*-abienol in the culture media

We assessed the stability of *cis*-abienol in the medium without the involvement of the strain, to determine that the degradation products were exclusively generated by the T2L strain's action. Using UPLC, we measured the initial concentration (0h), the concentration during fermentation (48h), and the final concentration (96h) of *cis*-abienol in the medium. It was observed that there were no significant changes in the *cis*-abienol content, indicating its stable presence in the medium. Consequently, we concluded that all degradation products were produced by the T2L strain's activity. Additionally, the pH of the medium at this point was 7.


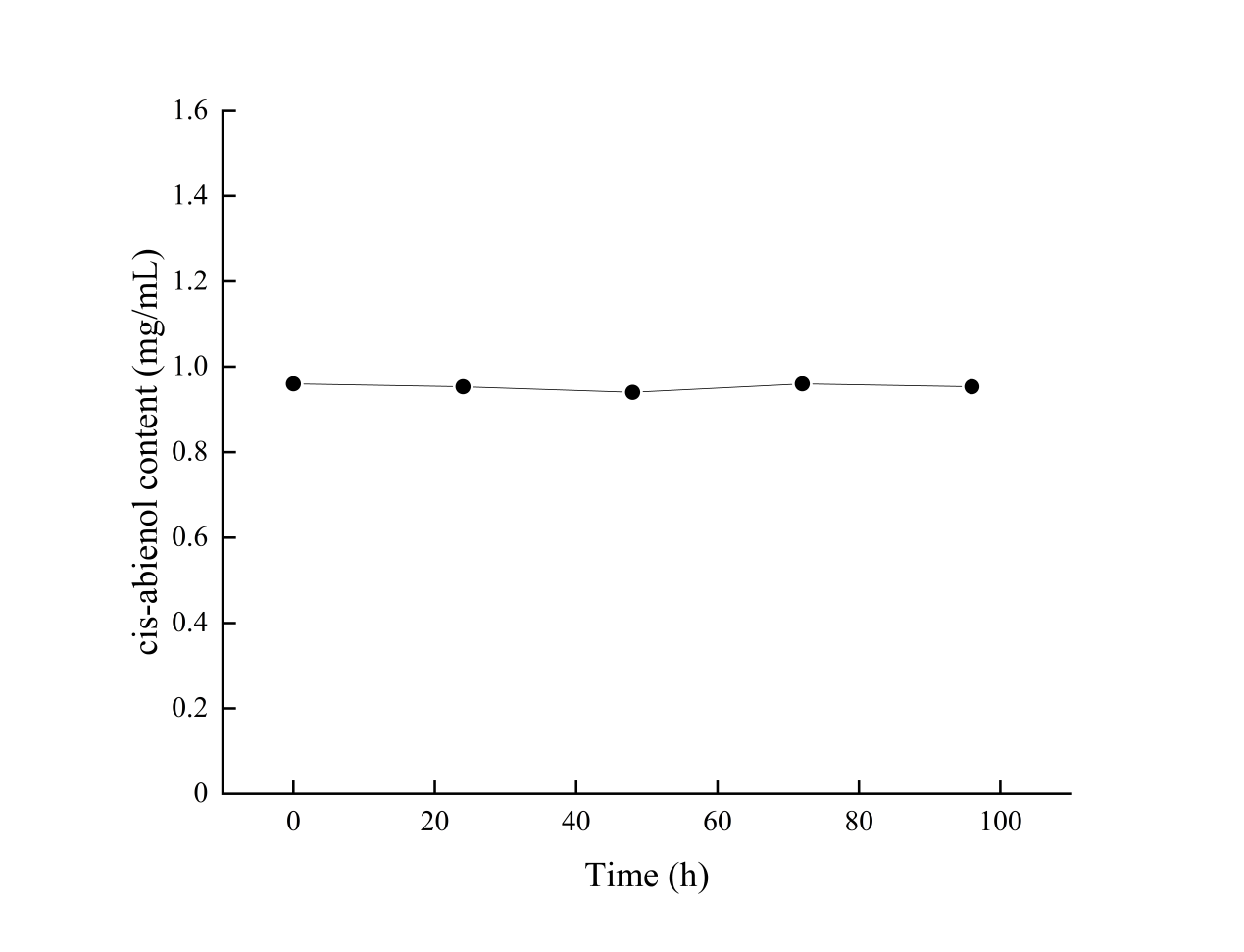


Figure S1. Stability of *cis*-abienol in culture media

1. Influence of varing pH value on *cis*-abienol in culture medium

Through investigating the impact of pH on the degradation efficiency of *cis*-abienol, it was discovered that the degradation rate slightly increased at a pH of 9.0. To further explore this, we studied the changes in *cis*-abienol content in the medium at both pH 9 and pH 5, without the addition of the strain. Utilizing UPLC analysis, we found no significant variations in the *cis*-abienol content at the initial (0h), intermediate (48h), and final (96h) stages of fermentation. Based on these observations, we can confidently attribute these findings to the action of strain T2L.
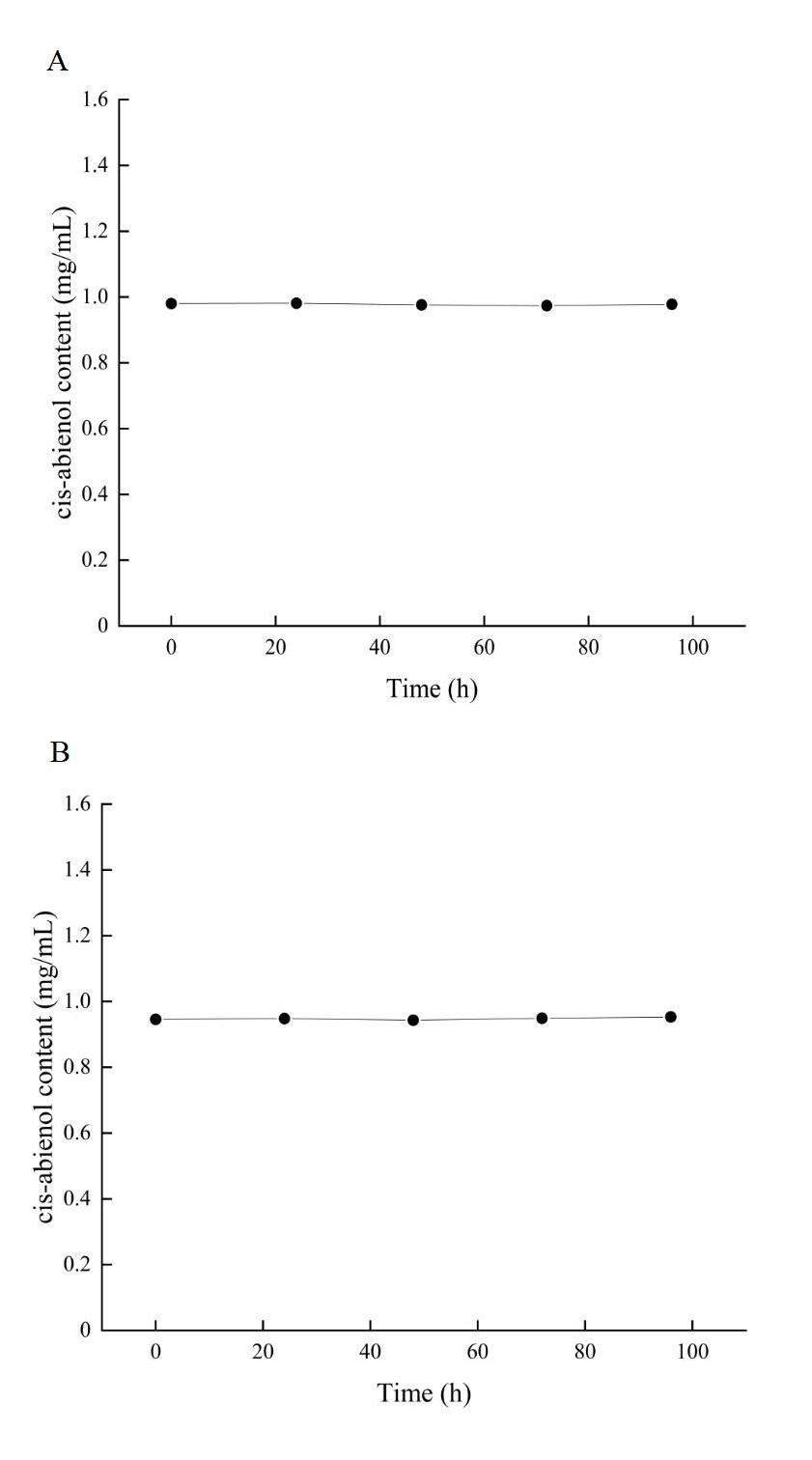


Figure S2. Effect of pH value on *cis*-abienol in culture medium with different pH. A. pH 5; B. pH 9

1. Monitoring of pH value during fermentation

During the cultivation of strains, real-time pH monitoring was not implemented, and in the comparative experiments involving different pH values, we solely observed the impact of the initial pH on the strains' response. To supplement this, we conducted an additional experiment where we selected a single carbon source (glucose) and two nitrogen sources (ammonium sulfate and peptone). We tested the pH value throughout the fermentation process and compared pH readings at different times of the fermentation. Our results revealed that there were no significant changes in the pH value during the entire fermentation process.


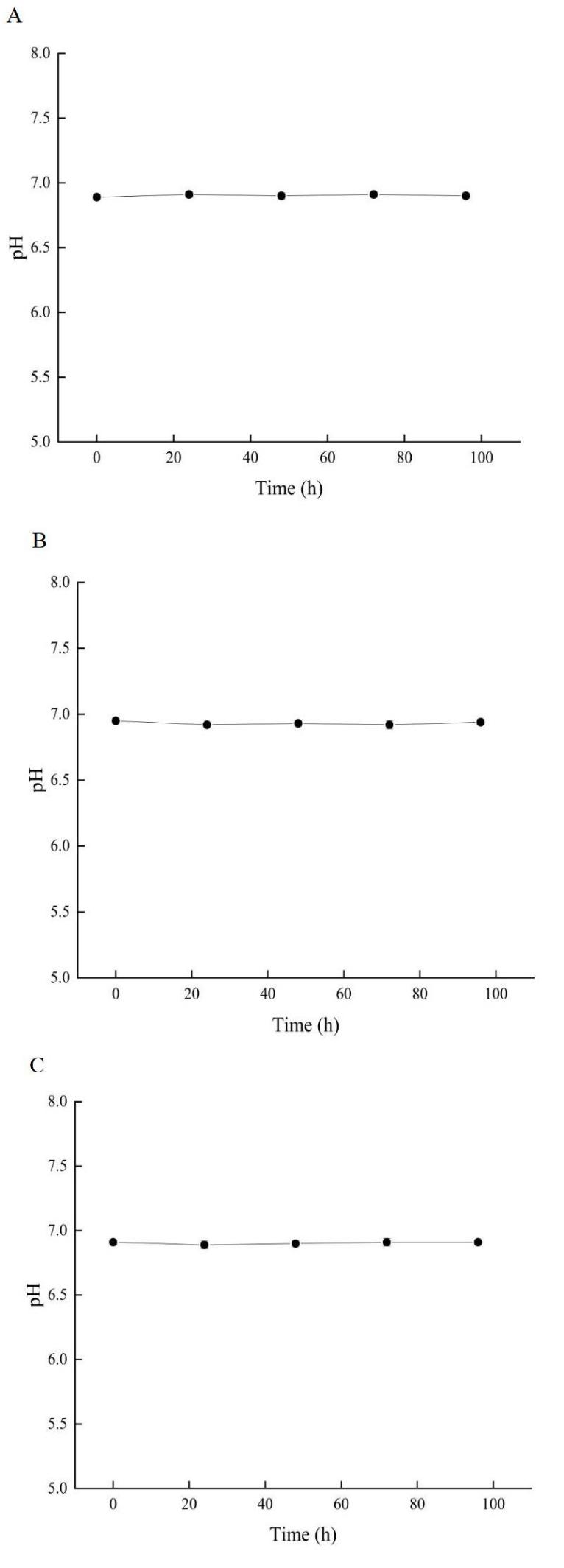


Figure S3. pH analysis of the whole fermentation process with different substances in the medium using T2L. A. Glucose; B. Ammonium sulfate; C. Peptone.

1. GC-MS analysis results of the compounds in Figure 4

All GC-MS data were compared by NIST 20.L spectral library

**Compound 1: n-Nonenylsuccinic anhydride**


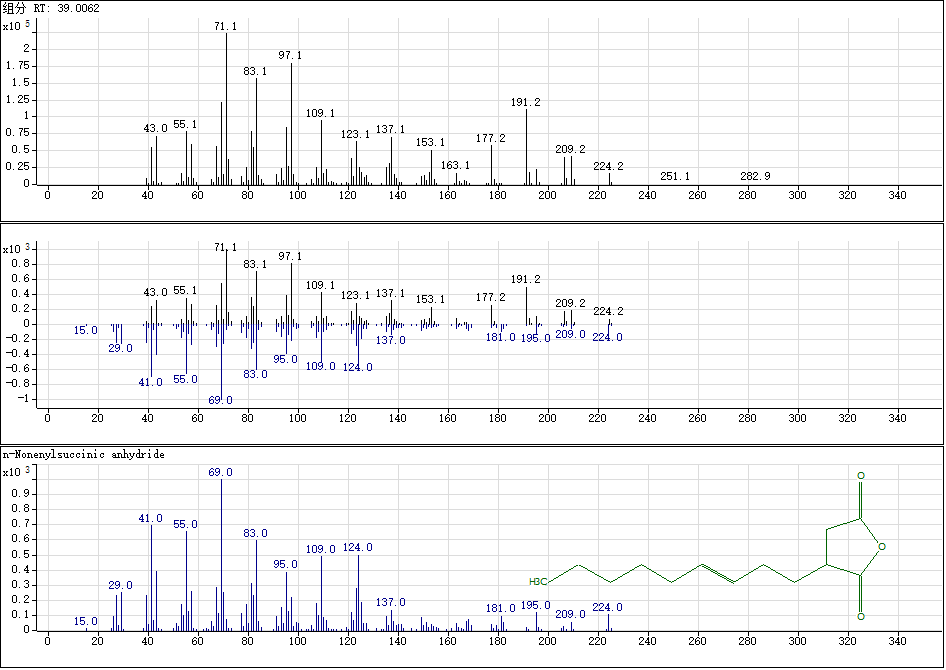


**Compound 2：Sclaral (sclareolide lactol)**


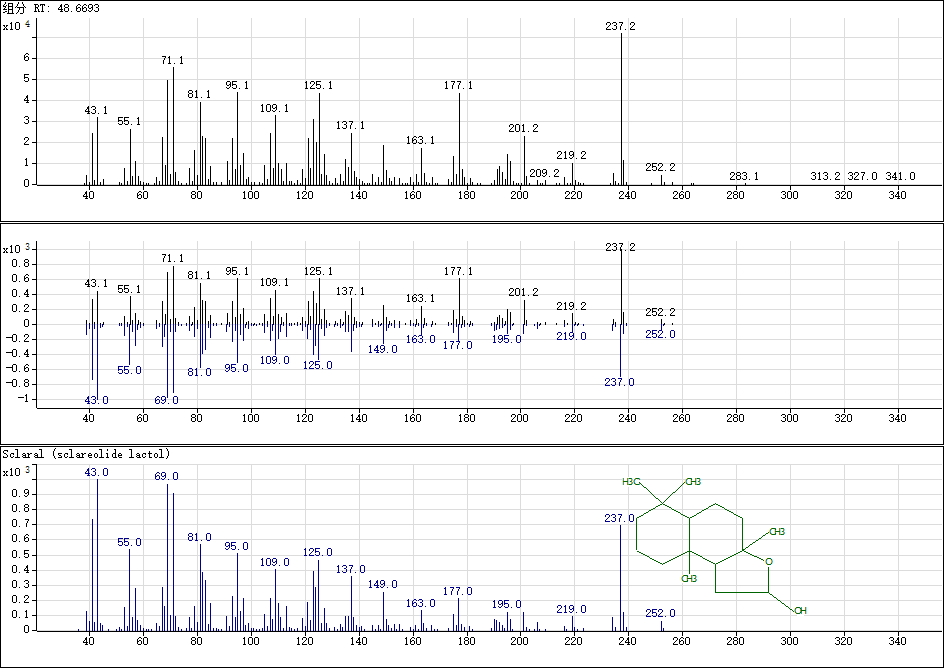


**Compound3：Amberonne (isomer3) analog**


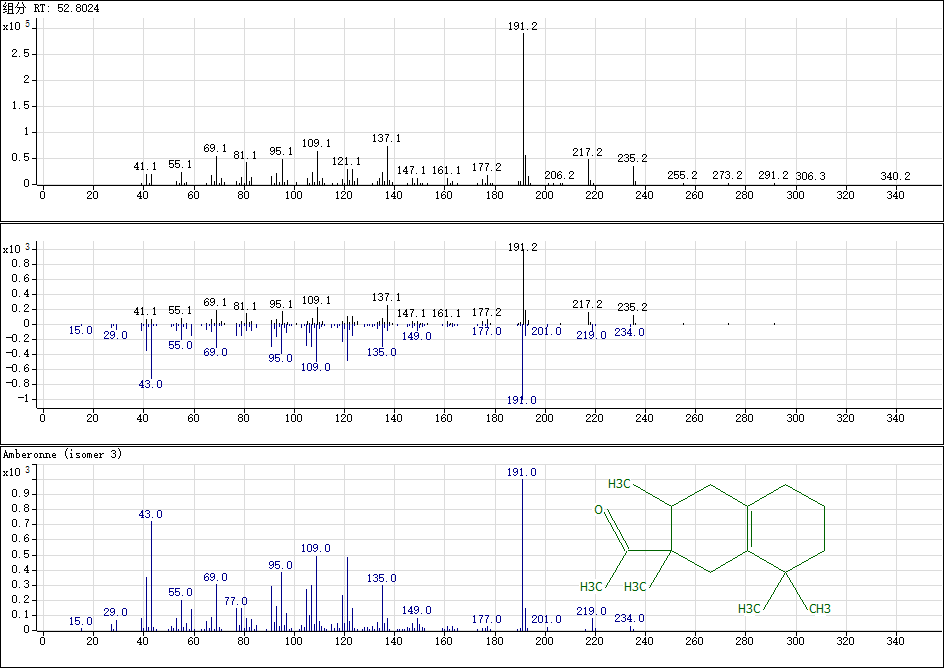


**Compound 4：**[**Sclareol**](https://www.chemsrc.com/en/cas/515-03-7_79307.html) **analog**


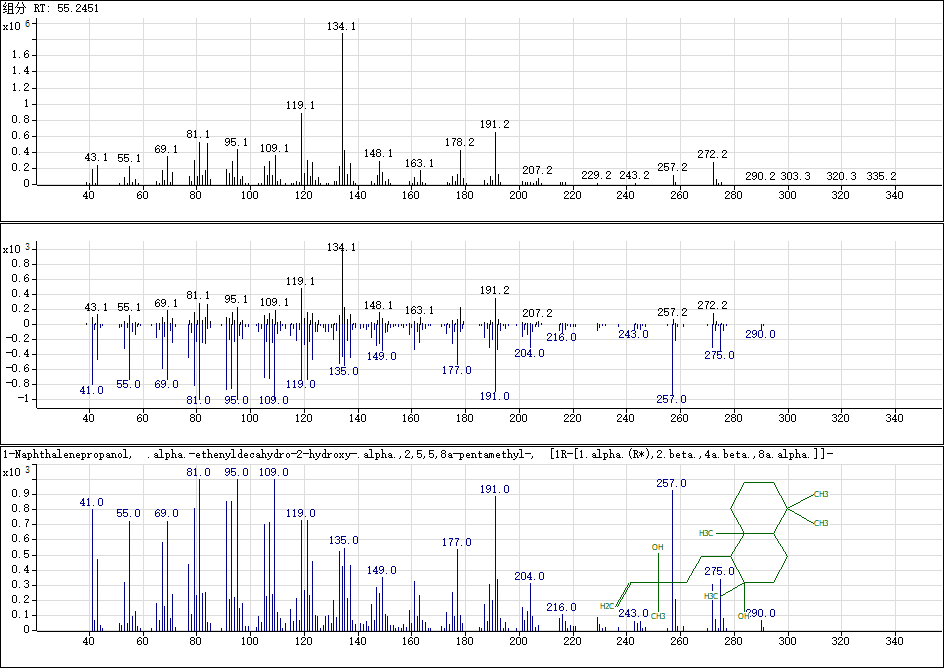


**Compound 5：15-Isobutyl-(13.alpha.H)-isocopalane**


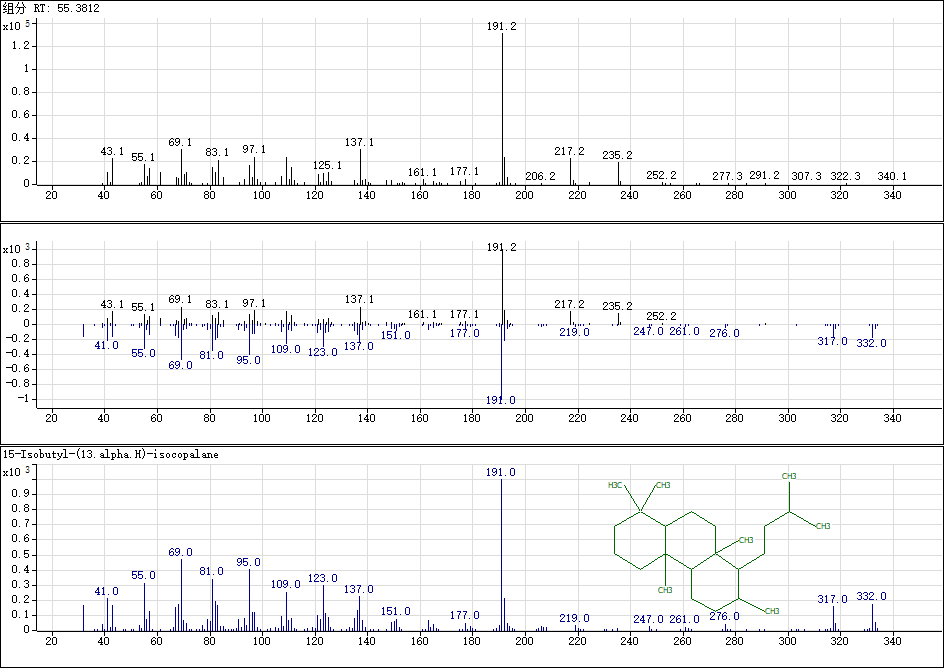


**Compound 6：(3R)-3,7-Dihydroxy-9,11-eremophiladien-8-one, 3-acetate**


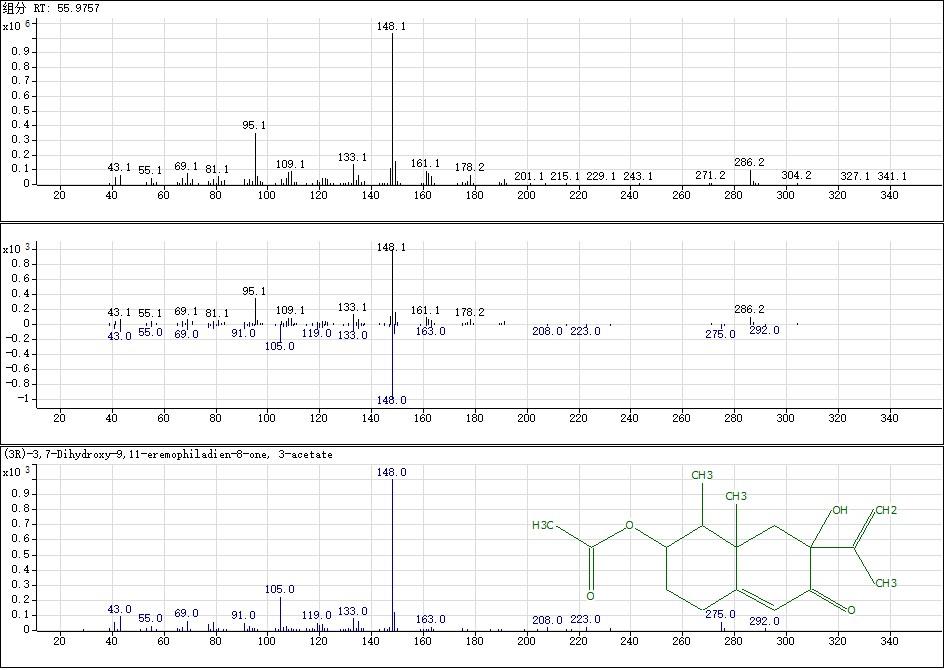


**Compound 7：2,5,5,8a-Tetramethyl-4-methylene-4a,5,6,7,8,8a-hexahydro-4H -chromene**


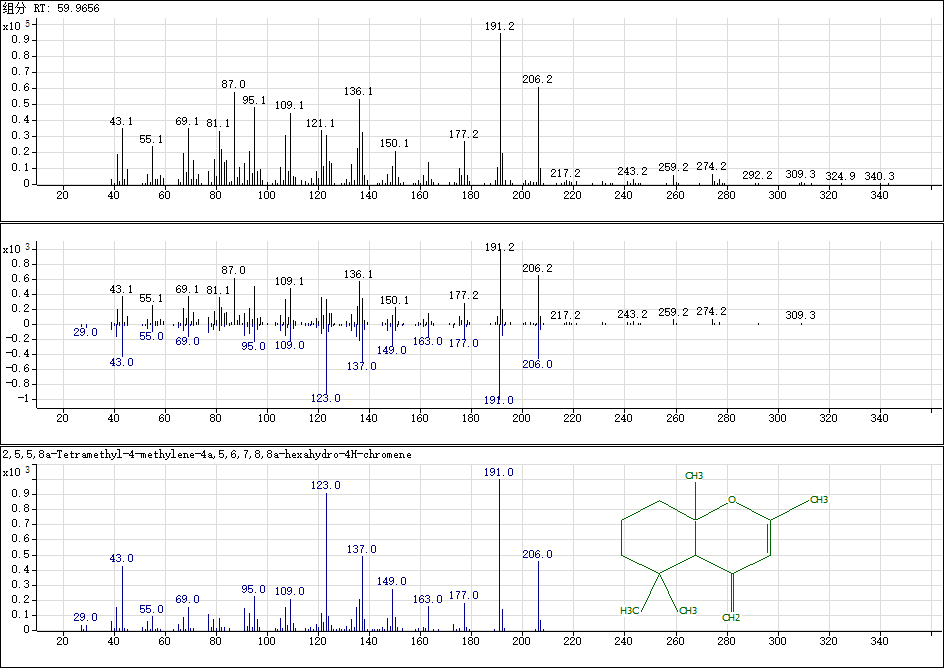


**Compound 8：Ambreinolide**


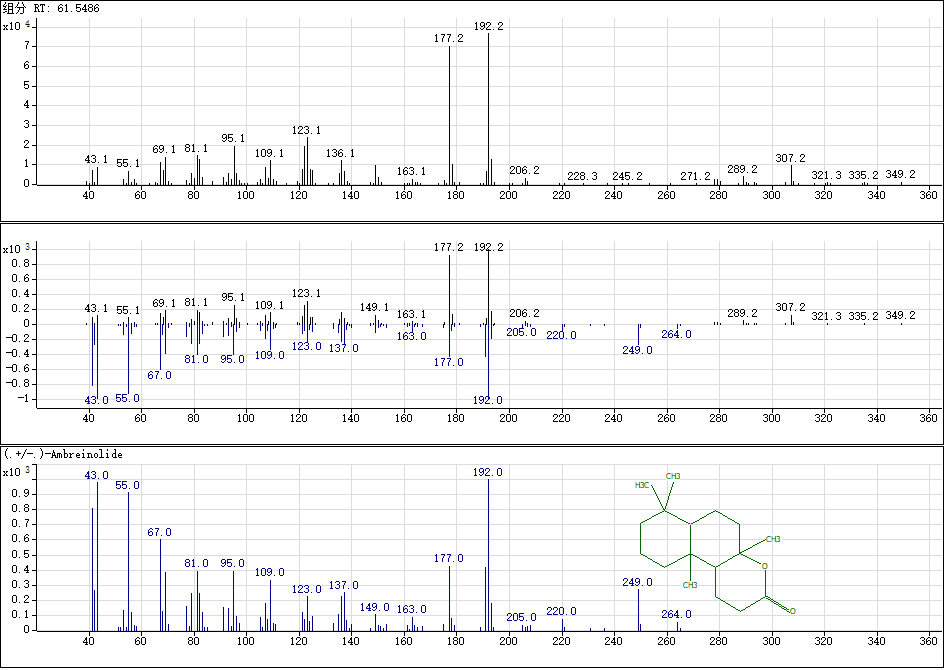


Figure S4. Peak ion spectra profile of the cis-abienol degraded products by GC-MS.
